# Fuzzy Risk-Based Maintenance Strategy with Safety Considerations for the Mining Industry

**DOI:** 10.3390/s22020441

**Published:** 2022-01-07

**Authors:** Agnieszka Tubis, Sylwia Werbińska-Wojciechowska, Pawel Sliwinski, Radoslaw Zimroz

**Affiliations:** 1 Faculty of Mechanical Engineering, Wroclaw University of Science and Technology, Wyspianskiego 27, 50-370 Wroclaw, Poland; agnieszka.tubis@pwr.edu.pl; 2 KGHM Polska Miedź S.A., M. Skłodowskiej-Curie 48, 59-301 Lubin, Poland; pawel.sliwinski@kghm.com; 3 Faculty of GeoEngineering Mining and Geology, Wroclaw University of Science and Technology, Wyspianskiego 27, 50-370 Wroclaw, Poland; radoslaw.zimroz@pwr.edu.pl

**Keywords:** system maintenance, risk, safety, RBM concept, fuzzy logic, mining industry

## Abstract

Enterprises today are increasingly seeking maintenance management strategies to ensure that their machines run faultlessly. This problem is particularly relevant in the mining sector, due to the demanding working conditions of underground mines and machines and equipment-operating regimes. Therefore, in this article, the authors proposed a new approach to mining machinery maintenance management, based on the concept of risk-based maintenance (RBM) and taking into account safety issues. The proposed method includes five levels of analysis, of which the first level focuses on hazard analysis, while the next three are connected with a risk evaluation. The final level relates to determining the RBM recommendations. The recommendations are defined in relation to the three main improvement areas: maintenance, safety, and resource availability/allocation. The proposed approach is based on the use of fuzzy logic. To present the possibilities of implementing our method, a case study covering the operation of selected mining machinery in a selected Polish underground mine is presented. In the case of mining machinery, fourteen adverse-event scenarios were identified and investigated; general recommendations were also given. The authors have also indicated further directions of research work to optimize system maintenance strategies, based on the concept of risk-based maintenance. Additionally, the discussion about the implementation possibilities of the approach developed herein is provided.

## 1. Introduction

The role of risk management is increasingly important in both the industrial arena and science as a whole.

One of the application areas of risk assessment regarding asset-management system improvement is the concept of risk-based inspection and maintenance (RBIM). RBIM can be used to identify critical equipment where inspections will provide the most benefit in reducing the overall risk [1]. This approach was primarily developed for the needs of the oil and gas sector, both for offshore and onshore facilities [2]. Currently, the approach is gaining popularity and is being implemented in other industrial sectors as well. Thanks to its implementation, the frequency of failures (mainly those requiring the long-term shutdown of the installation/equipment) is minimized, and the consequences of their occurrence are reduced. This concept is particularly welcome in those sectors of the economy where a higher standard of safety is required to operate the mechanical equipment and installations used. One of these sectors is the mining industry.

Mines, as complex human engineering systems, are often subject to multifaceted and multidimensional hazards. The effects of such hazards are usually associated with the loss of human life and health. Additionally, these impacts have affected both mineworkers and the environment, including the inhabitants of the areas adjacent to the mine. This is one of the reasons why risk management in the mining sector has become increasingly important in recent years. This is also confirmed by many reports that have been prepared for the mining sector, which focus on risk management in mines.

The growing importance of risk management in mining processes is also indicated by commercial reports developed for the management of the mining sector. One such report is the *Mining Risk Review*, published by Willis Towers Watson. This report has been published periodically since 2016. Each report focuses on a different risk issue in terms of emerging challenges and risks in the mining sector. The application of risk management concepts in the mining sector is presented in detail in [3]. According to the conducted literature review, one of the critical topics described in recent publications is the safe operation of mine machinery and technical equipment. Therefore, the RBIM approach is a highly essential concept applied in mining machinery operations.

Following this background, this article aims to present a new approach to maintenance management, based on applying the risk-based maintenance concept to the mining sector, taking into account the risk assessment of adverse event scenarios associated with the appearance of a hazard as a result of a mining machine failure. Accordingly, the proposed 5-level risk assessment method is based on the scenario approach proposed by Kaplan and Garrick [4] and the implementation of fuzzy logic.

Moreover, the importance of ensuring safety in the mining sector has been highlighted in many available scientific works (see, e.g., [5,6,7]). At the same time, there is a lack of studies that consider the safety aspect in the area of RBM application. The known works focus on safety culture investments [8]. As a result, the proposed approach considers the safety decision-making problem by indicating appropriate recommendations in this area, based on risk evaluation results. Additionally, the consequences of the occurrence of unwanted scenarios have been determined, from the perspective of process safety.

Maintenance and safety issue management should also include tasks related to resource availability/allocation. One of the possible areas for ensuring or improving maintenance management efficiency may be sharing maintenance resources, especially in those systems where maintenance resources are limited and stochastic maintenance tasks conflict with each other, which increases equipment downtime. Therefore, the proposed approach also includes this aspect in the decision-making process.

Moreover, the developed assessment method’s implementation possibilities are based on the example of a selected mining machine operating in a selected Polish mine.

**Figure 1:**
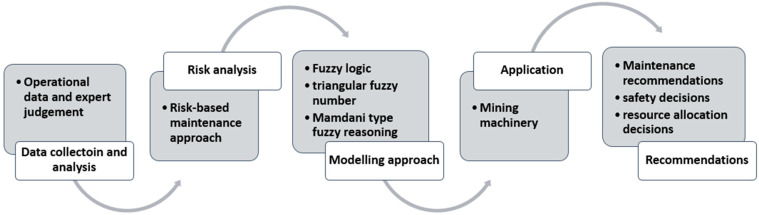
A framework of the developed decision-making solution.

To sum up, the framework of the developed solution is presented in Figure 1, and the authors’ contribution to this study includes:

- Introduction of the risk-based maintenance concept, based on risk assessment for possible consequences scenarios for mining machinery;
- Introduction of a risk-based maintenance strategy, based on safety aspects and resources availability/allocation issues regarding mining operation performance;
- Development of a five-step decision-making method to support mining machine maintenance planning processes;
- Implementation of fuzzy logic for scenario evaluations of consequences;
- Introduction of safety factors for severity/consequences assessment;
- Development of fuzzy RBM, based on a triangular fuzzy number that provides us with a beneficial way of maintenance decision-making in a fuzzy environment;
- Finally, the developed five-level decision-making method is implemented to verify the proposed method’s diagnostic function and determine its labor intensity.

Therefore, apart from the Introduction, the structure of this article includes a comprehensive review of the literature in the area of risk-based maintenance, taking into account fuzzy logic implementation and mining industry applications. Next, a proposed new approach to risk-based maintenance is presented. Moreover, the algorithm for risk assessment and estimation is described in detail. Later, in Section 4, the authors offer various implementation possibilities of the developed method. Section 5 presents the obtained results and a discussion of their implications. Finally, Section 6 provides conclusions, the limitations of the study, and suggestions for the authors’ future research work, to optimize a maintenance strategy based on the concept of risk-based maintenance.

## 2. Related Work

Over the last decades, maintenance has undergone a profound transformation, moving from a corrective to a proactive or predictive approach [9]. The need for this transformation stems from the fact that maintenance decisions nowadays increasingly concern complex industrial systems, and significantly affect the competitiveness and productivity of a company. Therefore, the relevance of maintenance and dependability analyses increased due to their role in improving availability, performance efficiency, product quality, timely delivery, environment and safety requirements, and total plant cost-effectiveness at high levels [10]. Simultaneously, the development of maintenance strategies is because companies need occupational health and safety management systems that help them prevent and mitigate accidents, by identifying and selecting the most essential hazards and by managing threats and their preventive measures [11]. Therefore, the risk of anticipated adverse events has become a cornerstone of the decision-making process of service managers [12]. This is supported by the research of Backlund and Hannu [13]. Effective use of resources can be achieved by using risk-based maintenance decisions to determine where and when to perform maintenance. The concept of risk is used in this case to measure the potential of losses caused by human activities and technical systems, together with the environment [11,14].

Risk-based maintenance is a concept based on risk in the area of technical systems management. The RBM concept emerged in the 1990s and provided a new vision for asset integrity management [13]. RBM approaches minimize the probability of system failure while mitigating its consequences via integrating maintenance activities with safety issues [15]. This methodology provides a tool for maintenance planning and decision-making. The RBM strategy uses each risk level as a criterion to plan maintenance tasks [16]. The most significant maintenance efforts and costs should be dedicated to high- and medium-risk scenarios.

Conversely, for low-risk adverse events, the scope of work and financial effort should be reduced in a structured and justifiable way. The quantitative value of the risk is used to prioritize inspection and maintenance activities. RBM suggests a set of recommendations regarding how many preventive tasks (including the type, means, and timing) are to be performed [2]. The resulting maintenance program aims to maximize the reliability of the equipment and minimize the cost of the total maintenance cost [17].

**Figure 2:**
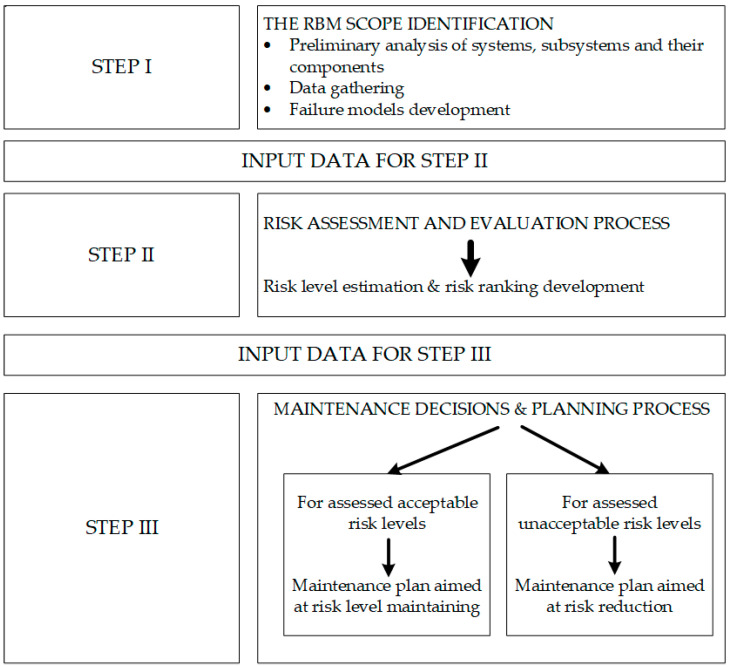
The architecture of RBM strategy. Source: own contribution based on [17].

According to the concept presented by [2], RBM consists of three basic modules: (1) risk determination, which consists of risk identification and estimation; (2) risk evaluation, which consists of risk acceptance analysis; and (3) maintenance planning, considering risk factors. The detailed analytical procedure is shown in Figure 2.

Additionally, three main approaches to risk analysis and assessment can be defined according to [14]—quantitative, qualitative, and hybrid approaches.

A summary of risk analysis methodologies classification has also been introduced, e.g., by the authors of [15].

In one study [18], the author emphasizes that risk assessment in RBM should include such phases as hazard identification, the evaluation of so-called preventive safety measures, along with their functionality, the estimation of exposure to existing hazards, and the assessment of all consequences. These consequences are usually risks and economic losses. In addition, it is important to remember that there are three key characteristics of risk assessment [16]: impact on human safety, environmental risk, and economic loss. It should also be remembered that economic losses can be expressed in terms of value, which is difficult in the case of evaluating personnel injuries or environmental degradation.

The RBM approach has been successfully used for maintenance decisions regarding mechanical and civil infrastructure, onshore and offshore structures, and cross-country pipelines [19]. In particular, it is used in sectors that are considered to be hazardous, such as, e.g., the oil and gas sector [9,20,21,22] or the energy sector, including the maintenance of complex systems in power plants [17,23,24,25]. An example of risk-based predictive maintenance for safety-critical systems is presented in [26]. Human error probability implementation for risk-based maintenance strategy development is introduced by the authors of [27]. An illustrative example of RBI implementation for physical asset inspection frequency optimization is given in [28]. A significant number of publications also deal with the application of RBM in the transport sector. The failure of a vehicle, particularly in terms of public transport or the transport infrastructure, results in a risk to health and life for many travelers. Examples of such studies are publications on the application of RBM in terms of tram maintenance [29], vessels and warships [30], and road infrastructure [31,32]. It should be noted that since the year 2000, the terms risk-based inspection (RBI) and risk-based maintenance (RBM) have been used interchangeably [19]. Because both terminologies refer to the same set of actions, risk-based maintenance, and risk-based inspections are no longer considered separate topics [33].

Despite many works available in the literature that focus on risk assessment for the mining industry (see, e.g., [34] as an example, or [3] for a comprehensive review), only a few publications deal with the application of RBM in the mining sector. A literature review conducted according to the keywords “risk-based maintenance + mine” identified only a few publications, of which the following are the most relevant [35,36,37,38]. The authors of [35] introduce a maintenance priority methodology for system components, based on a reliability allocation approach. The introduced methodology is applied for draglines operating in the Tuncbilek coal mine in Turkey. The next work [36] focuses on surface exploitation and coil refinement in the Serbian mining industry. They investigate the risk assessment problem in relation to dependability, safety, and maintenance management of the mining technological systems. In another work [37], the authors focus on maintenance strategy selection for draglines, based on AHP approach use. The case study is based on the operational data from a coal mine located in Northern India. The last work [38] focuses on the RBM planning conducted on load haul dumpers (LHDs) operating in Sindesar Khurd Mine, an underground zinc and lead mine situated in Dariba Rajasthan, India.

Additionally, primary maintenance decisions are subject to uncertainty related, among other things, to uncertainty in the data, errors in the reasoning process, or the occurrence of human error [39]. This problem is also connected with selecting the best maintenance strategy for technical systems [9]. Following the identification of this issue, many methods were developed for understanding, modeling, and managing uncertainty in risk, reliability, and maintenance. One of these approaches is fuzzy logic.

Fuzzy logic is based on the theory of fuzzy sets, developed and introduced by Zadeh [40]. This approach effectively uses flexible reasoning and considers inaccuracy, subjectivity, uncertainty, and imprecision [41]. Additionally, fuzzy sets theory is a convenient solution for situations where there is no conventional model available for estimating and measuring or if the model is too complex [42].

Fuzzy logic is widely implemented for risk and reliability assessments (see, e.g., [43,44]. One of the fundamental maintenance problems in which fuzzy logic has been applied is maintenance selection, especially perceived as a complex multi-criteria decision-making process (see, e.g., [45,46]). The scope of problems solved using fuzzy logic in this area is presented in Table 1. There are also a number of research works focused on the mining sector. The fuzzy approach is implemented in [47], where the authors introduce fuzzy FMEA for load-haul-dumper (LHD) risk assessment. Case studies for fuzzy risk assessment are presented, e.g., in [48,49]. In work [48], the authors focus on the performance of underground coal mines in Turkey, whereas in [49], the authors focus on a pebble crusher operating in a platinum-mining company. A hybrid fuzzy multi-criteria decision-making model for supplier selection regarding the mining sector is presented in [50]. Fuzzy risk assessment based on the fuzzy reasoning approach (FRA) and the fuzzy analytic hierarchy process (AHP) process is introduced in [51]. Risk assessment for fatal accidents in underground coal mines, based on FRA, is also presented in [52].

A risk assessment model for belt conveyor elements operating in a coal mine is introduced in [53]. The authors implement fuzzy sets theory, fuzzy logic, and min-max composition for the risk assessment of mining equipment failures. Fuzzy expert assessment is implemented by the authors of [54] for machine-failure severity evaluation.

A short summary of the available literature on fuzzy logic used in the area of RBMI modeling is presented in Table 1.

To sum up, several maintenance approaches and different tools can be distinguished for the proper selection and definition of maintenance strategy. Therefore, the problem of selecting the most-suited maintenance policy can be perceived as a difficult task deeply affected by the maintenance skills and available resources inside a given organization. Additionally, as a high-risk sector, maintenance strategies should also include safety aspects for the mining industry. Following the conducted literature review, there is still a space for the development of RBM methodologies for the mining sector.

Therefore, our proposed solution is based on RBM approach implementation to support a decision-maker in maintenance, safety, and resource allocation decisions.

**Table 1:** Summary of reviewed literature on fuzzy RBMI.

Ref. No.	Problem Investigated	The Main Goal of the Implemented Approach	Type of Paper	Risk Analysis Methodology Implemented	Modeling Method Used	Case Study
[55]	The fuzzy logic decision process for planning the maintenance activities	Maintenance interval planning	Research paper	n/a	Fuzzy logic, statistical testing, signal processing	Cold plastic deformation tools
[47]	Comparison of fuzzy FMEA and conventional FMEA results	Potential failure mode ranking (risk prioritization)	Case study	Fuzzy FMEA	Fuzzy logic	LHD machine
[41]	Assessment of maintenance failure risk			Risk matrix method		LPG supply chain
[48]	Comparison of conventional risk matrix method and fuzzy risk assessment		Research paper			Underground coal mine
[53]	Comparison of conventional FMEA method and fuzzy risk assessment			FMEA	Fuzzy sets theory, fuzzy logic, and min–max composition	
[45]	Maintenance policy selection and comparison with results obtained using conventional AHP with goal Programming	Selection of the best suitable maintenance policy	Research paper	n/a	Fuzzy ANP	Chemical plant
[50]	Multi-criteria decision-making problem	Supplier selection for forklift filters	Research paper	n/a	Fuzzy AHP, fuzzy DEMATEL and TOPSIS	Mining company
[51]	New model for evaluation of risk levels associated with identified hazard factors in mining industry	Mine lever risk estimation			Fuzzy reasoning approach and fuzzy AHP	Metalliferous mine
[49]	Crushing circuit optimization	Determining the major crusher failures	Case study	Root Cause Analysis	Fuzzy logic, stresses analysis	Platinum mining company
[54]	Risk assessment model development for mining machinery	Failure severity assessment	Research paper	FMEA	Fuzzy sets theory, fuzzy logic, and min–max composition	Mobile crushing machine
[39]	Fuzzy RBM model development	Fuzzy risk-index evaluation		Risk decision matrix	Delphi method, fuzzy logic	Oil and gas refineries
[56]	RBM optimization	Functional failure risk of equipment prioritization			Fuzzy set theory and fuzzy logic	Offshore oil and gas production and process industry
[57]		Risk rank calculation	Case study		Fuzzy logic	Manufacturing company
[52]	Basic fuzzy RBM model application	Fuzzy risk-index evaluation				Underground coal mine
[46]	Risk-based maintenance plan definition	Risk-index optimization	Research paper	FTA, ETA	HAZOP, fuzzy AHP, bi-objective fuzzy structure optimization modeling	Offshore processing facility
[58]	Reliability allocation method development; comparison of traditional RPN-based and fuzzy allocation methods	Reliability and cost coefficients estimation	Research paper	n/a	Fuzzy logic	Spindle system of numerical control machine
[43]	Multi-state performance reliability model development	System reliability estimation	Research paper	n/a	Fuzzy set theory, probability theory, simulation modeling	Harmonic gear reducer
[44]	Prediction of equipment failure onboard tankers	Failure modes prioritization		Fuzzy FMEA	Fuzzy set theory, grey theory	Tanker equipment

## 3. The Concept of Fuzzy Risk-Based Maintenance Strategy

**Figure 3:**
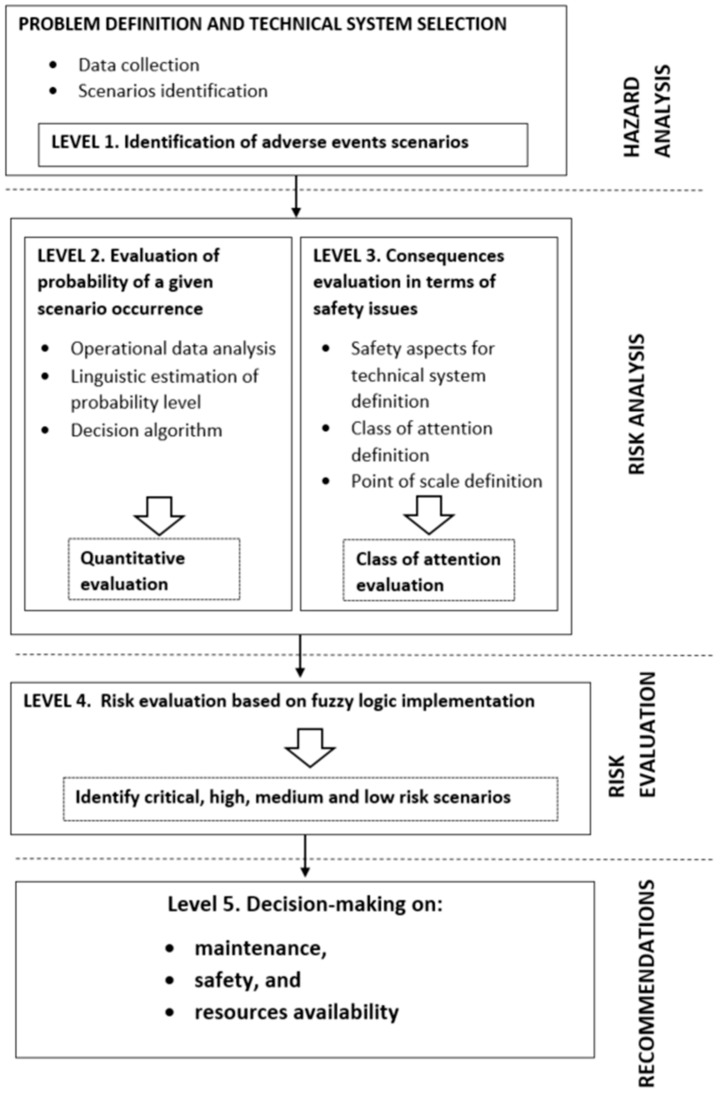
Fuzzy RBM methodology, as proposed in this study.

The proposed method for maintenance strategy definition assumes five levels of analysis, of which the first four levels focus on risk assessment and evaluation, while the last one relates to the determination of the scope of maintenance activities undertaken, in relation to safety issues and resources availability. A detailed scheme of the procedure is presented in Figure 3. A detailed discussion of the relevant phases of the proposed procedure is outlined in the next subsections.

Additionally, the proposed solution is based on the fuzzy logic approach with the use of triangular fuzzy numbers. This requires a theoretical background to be provided in this area.

### 3.1. Preliminaries

First, the concepts of fuzzy sets and triangular and trapezoidal fuzzy numbers are briefly reviewed. A detailed review can be found, e.g., in [40,59].

**Definition** **1** ([60])**.** *Let us assume that X is a space of points with a generic element of X denoted by x. Thus, X = {x}. In addition, let us assume that the X element belongs partially to set A and partly to its complement. Each set X element has a defined value that characterizes the degree of membership to the fuzzy set. Following that the standard fuzzy sets membership function belongs to a range [α, β], the membership function of the normal set X is:*

(1) $$\mu_{A}:X\to \left[0,1\right]$$

**Definition** **2** ([60])**.** *A fuzzy set A is contained in fuzzy set B only when* $\mu_{A}\left(x\right)<\mu_{B}\left(x\right)$ *for each* x∈X *, and the fuzzy set A equals fuzzy set B only when* $\mu_{A}\left(x\right)=\mu_{B}\left(x\right)$*. The complement of set A is a fuzzy set* $\bar{A}$ *with a membership function* $\mu_{\bar{A}}=1-\mu_{A}$.

**Definition** **3** ([61,62])**.** *A triangular fuzzy number (FN), A_z_ = (a, b, c) is a fuzzy set defined as the set, R, of real numbers, whose membership function is given by:*

(2) $$\mu_{z}\left(x\right)=\{\begin{array}{c} 0\;\;\;for\;x<a\\ \begin{array}{c} \frac{x-a}{b-a}\;\;\;\;for\;a\leq x\leq b\\ \frac{c-x}{c-b}\;\;\;\;for\;b\leq x\leq c\\ 0\;\;\;for\;x>c \end{array} \end{array}$$

*The FN parameters meaning is straightforward: a and c are the lower and upper bounds of fuzzy number A_z_, respectively, and b denotes the modal value of fuzzy number A_z_. If a ≥ 0, then an FN A_z_ is called a positive triangular FN. If c ≤ 0, then an FN A_z_ is called a negative triangular FN.*

**Definition** **4** ([63])**.** *A trapezoidal fuzzy number A_z_ = (a, b, c, d) is a fuzzy set defined on the set, R, of real numbers, whose membership function is given by:*

(3) $$\mu_{z}\left(x\right)=\{\begin{array}{c} 0\;\;\;for\;x\leq a\\ \begin{array}{c} \frac{x-a}{b-a}\;\;\;\;for\;a\leq x\leq b\\ 1\;\;\;\;\;\;\;for\;b\leq x\leq c\\ \frac{d-x}{d-c}\;\;\;\;for\;c\leq x\leq d\\ 0\;\;\;\;\;\;\;\;\;\;\;for\;x\geq d \end{array} \end{array}$$

### 3.2. The Main Phases of the Proposed Fuzzy RBM Methodology

**Level 1: Identification of adverse event scenarios**

The first level of the analysis is to identify undesired events that may occur in connection with the operation process of the analyzed mining machines. Their description is in the form of scenarios, as the proposed method uses in the risk assessment the scenario approach proposed by Kaplan and Garrick [4]. Therefore, the risk is defined as:

(4) $$R=\left\{S_{i},\;P_{i},\;C_{i}\right\},\;\;\;\;\;\;\;\;\;i=1,\;2,\;\ldots ,\;N$$

where: *R*—risk; {}—must be interpreted as a “set of”; *S_i_*—*i*th scenario (undesirable event) description; *P_i_*—the probability of *i*th scenario occurrence; *C_i_*—the measure of consequences or damage caused by the *i*th scenario; *N*—the number of possible scenarios.

Based on historical data, service documentation, and benchmarking analysis, individual adverse event scenarios are identified that should be subject to further analysis in the opinion of managers. At this stage of the procedure, it is recommended to use selected qualitative methods that are described, among others, in the ISO 31000:2018 standard [64]. Based on the experience gained within the SafeMe4Mine project, among the recommended methods it is suggested to use: (1) the “*What*—*if*” method (SWIFT), (2) the check-list method, and (3) the HAZOP method. Prepared scenarios should primarily concern the failure of individual subsystems or critical elements of the machine.

**Level 2: Evaluation of the probability of a given scenario occurring**

The starting point for Level 2 analysis is the computerized maintenance management system handling the available data about the occurred failures and the conducted inspection/planned/corrective maintenance in the analyzed period. On this basis, it is possible to estimate the probability of occurrence of the identified adverse event scenarios.

**Figure 4:**
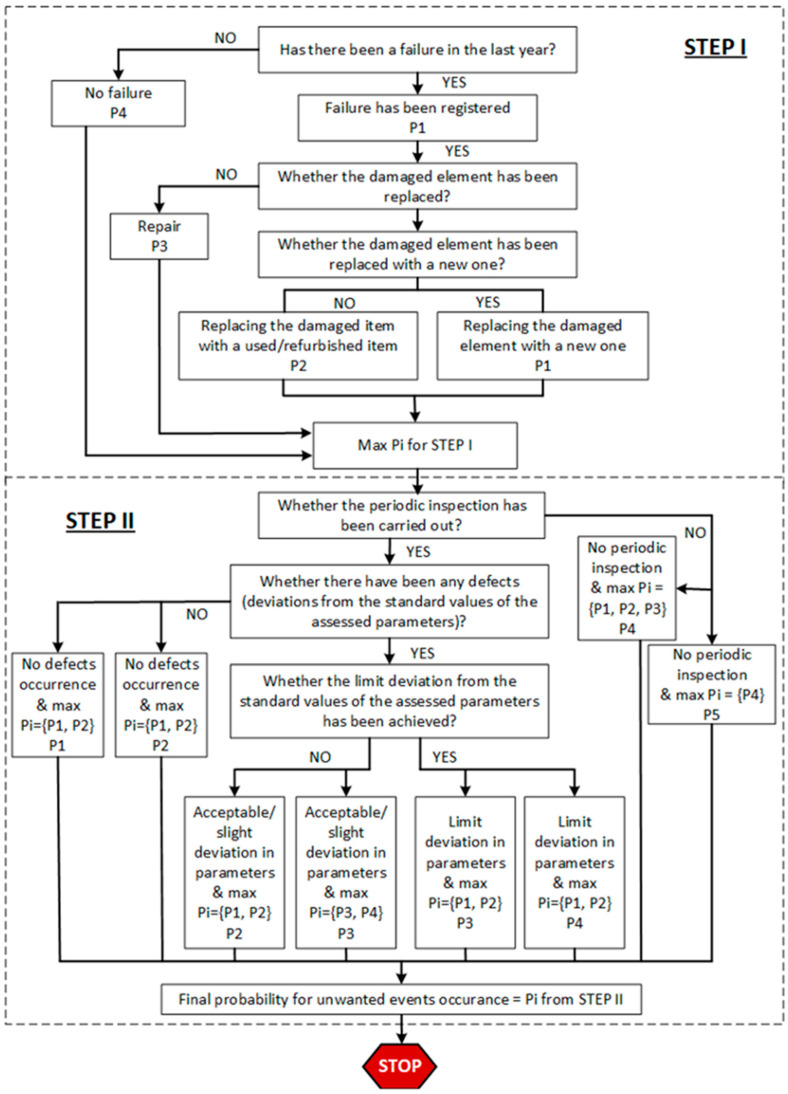
Algorithm for unwanted events occurrence probability estimation.

The probability of occurrence of a given scenario is estimated according to the algorithm presented in Figure 4. The procedure follows two steps and assumes that: (1) the lack of failure in the analyzed past period increases the probability of its occurrence in future periods; (2) the replacement of failed elements with a used/refurbished part or their so-called imperfect repair (e.g., minimal repair) increases the probability of their failure in future; (3) lack of results from the conducted periodic inspection after the last performed repair increases the probability of failure (lack of information increases the uncertainty of the decision made). The first step of the probability estimation is based on the failure history of a system/element. If there was no failure, the probability of a given scenario occurring should be estimated at the level P4 (Likely to happen in the near time). When the failure was identified and repaired, the probability estimation follows the P3 level (possible to occur in the near future). If the failed element was replaced by a new one, the Pi level equals P1, otherwise, it equals P2. The first step of the decision algorithm ends with the definition of max Pi level, which was estimated based on the reasoning process.

The second step is based on the results obtained from periodic inspection performance. Based on the results from the first step (max Pi) and the decision process from the second step, the final probability of the unwanted scenario occurrence is defined.

According to the decision algorithm, the probability can take levels from P1 to P5, with the following linguistic notation being assigned to the next levels (Table 2).

**Level 3: Class of attention definition**

The managers should determine the attention class defined for each scenario, based on the selected ranking criteria. In the mining sector, the determination of the attention class should take into account the potential consequences of an adverse event occurring, taking into account numerous safety aspects. In terms of process safety, consequences can be defined as damage to property or assets, human health loss, toxic release, environmental harm, damage to the company’s reputation, and production loss (see, e.g., [6,54,65]). In the study by [46], four major categories for consequence analysis are included: human loss, economic loss, environmental damage, and reputation. Personnel, equipment, operation, and environment are investigated in work [66]. Considering such approaches and based on mining sector conditions, in this study, the consequences are investigated in relation to the three main aspects—human loss, the time taken to take the machine out of service (downtime and production delay), and economic loss (including assets, property, production, and reputation). As a result, the definitions of the consequences in relation to the three main groups of effects, namely, human consequences, machinery, and costs, are presented in Table 3.

**Level 4: Risk evaluation based on fuzzy-logic use**

During this phase, the main steps are collecting expert opinions and implementing a fuzzy model to evaluate the risk ratio for all the identified unwanted scenarios. One potential issue to consider at this stage is the assessment of experts in terms of their knowledge, experience, and availability. On the one hand, the results obtained will allow assessing whether weighting factors for experts should be introduced. On the other hand, the results obtained may influence, e.g., the estimation of the optimum number of expert scores required to reach a judgment. This problem is considered, among others, in the work [66]. First, the experts provide their opinions for the defined risk assessment parameters. The experts’ opinions are collected using linguistic scales. The proper definition of linguistic input variables is based on expert knowledge and the description given in Table 2 and Table 3.

**Figure 5:**
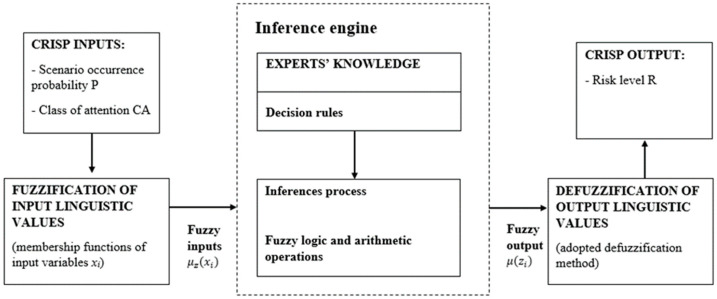
The structure of a typical fuzzy logic system (FLS) [39,48,57].

When the expert opinions are collected, the fuzzy set theory is used. According to the preliminaries defined in Section 3.1, the proposed solution is based on triangular FN use for input variables and trapezoidal FN for the output variable. Following Figure 5, the basic structure of the fuzzy logic system (FLS) includes three main parts: fuzzification, the fuzzy inference system (FIS), and defuzzification [56].

**Figure 6:**
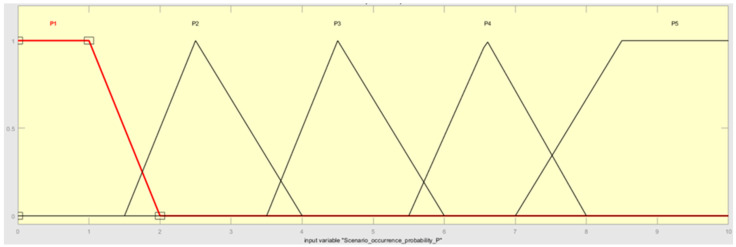
The scenario occurrence probability fuzzy sets.

**Figure 7:**
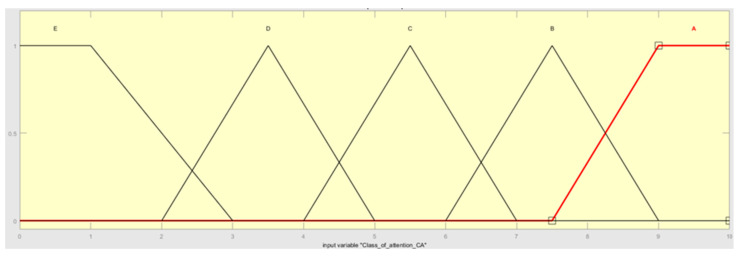
The class of attention fuzzy sets.

The first process connected with fuzzification decomposes input variables (scenario occurrence probability (P), class of attention (CA)), and one output variable (risk (R) categories) with crisp numbers into fuzzy sets (P, CA, R). In the current work, the authors decided to use triangular and trapezoidal FNs, due to their ease of application and versatility. The two input variables, P and CA, are described linguistically in Table 2 and Table 3. The fuzzy sets for variables P and CA are presented in Figure 6 and Figure 7.

**Figure 8:**
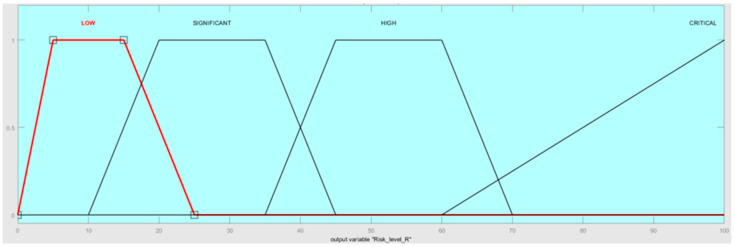
The risk categories’ fuzzy sets.

There are also defined four main categories for risk level. According to these categories, the risk is ranked as low (tolerable/acceptable), significant, high, and critical (intolerable/unacceptable). Risk fuzzy sets are conducted according to expert opinions, by our own analysis, and are presented in Figure 8. Table 4 shows the range of linguistic variables proposed for the decision-making process.

Usually, a single manager or engineer cannot consider all relevant aspects of an underground mine. Following this assumption, risk assessment in the mining sector involves experts with different backgrounds and experiences, who may have different opinions on the final judgment. As a result, it is necessary to aggregate all experts’ opinions to get an overall quantified value. According to [52], the aggregation of expert opinion can be performed using the arithmetic mean aggregation operator. The mean aggregation operator, defined in fuzzy triangular numbers (*a*_1_, *b*_1_, *c*_1_), (*a*_2_, *b*_2_, *c*_2_)… (*a_m_*, *b_m_*, *c_m_*), delivers the result as (*x*, *y*, *z*) according to the formula:

(5) $$\{\begin{array}{c} x=\frac{1}{m}\overset{m}{\underset{k=0}{\sum }}a_{k}\\ y=\frac{1}{m}\overset{m}{\underset{k=0}{\sum }}b_{k}\\ z=\frac{1}{m}\overset{m}{\underset{k=0}{\sum }}c_{k} \end{array}$$

In this paper, the authors do not consider expert weighting. However, in a scenario where it is possible to use the evaluation of experts with different levels of knowledge and experience, it is recommended to introduce weighting factors for their evaluation. More information about experts’ fuzzy opinions’ aggregation may be found, e.g., in [67,68].

The second part of the FLS is the fuzzy inference system. It maps fuzzy input tests into fuzzy output sets using a knowledge base [69]. The proposed solution is based on the Mamdani-type fuzzy engine and the “IF-THEN-ELSE” rules, established based on human knowledge and mathematical calculus. The definition of the If-Then rules based on the risk decision matrix is presented in Table 5. Following this method, 25 rules can be defined. According to this system, for example, rule 1 is defined as:

*IF Scenario occurrence probability is P1 and Class of attention is E, THEN Ri level is Low.*

**Table 5:** Risk decision matrix for “IF-THEN-ELSE” rules definition.

**Scenario Occurrence Probability**	**P5**	SIGNIFICANT	HIGH	HIGH	CRITICAL	CRITICAL
	**P4**	SIGNIFICANT	SIGNIFICANT	HIGH	CRITICAL	CRITICAL
	**P3**	LOW	SIGNIFICANT	SIGNIFICANT	HIGH	HIGH
	**P2**	LOW	SIGNIFICANT	SIGNIFICANT	SIGNIFICANT	HIGH
	**P1**	LOW	LOW	LOW	SIGNIFICANT	SIGNIFICANT
		**E**	**D**	**C**	**B**	**A**
		**Class of attention**				

Due to the use of the Mamdani model, the FIS was based on MIN and MAX operator implementation. The MIN operator was used for combination and implication operations. The MAX operator was used to aggregate the fuzzy outputs. Additionally, the MATLAB fuzzy logic toolbox was used to build a FIS for the RBM modeling.

The last part of FLS is the defuzzification process. During this process, the obtained fuzzy outputs into crisp values are based on a known defuzzification method. Widely used defuzzification methods include, among others, centroid average (CA), the center of gravity (COG), the mean of maximum (MOM), or largest of maximum (LOM). A survey of the most commonly known defuzzification methods is presented, e.g., in [70]. The authors propose to use a COG method due to its simplicity and providing a better solution than other methods. Thus, the crisp output is estimated as [52]:

(6) $$Centroid\;of\;area,\;z^{*}=\frac{\int \mu_{A}\left(z\right)\cdot zdz}{\int \mu_{A}\left(z\right)dz}$$

where: z*—the crisp value for the *z* output (defuzzified output); $\mu_{A}\left(z\right)$—the aggregated output membership function; *z*—the universe of discourse.

After the defuzzification process, the FIS gives a crisp output value to express the risk level of the associated hazard scenario occurrence. Following this, the proper actions can be ordered, based on the definition of recommendations.

**Level 5: Recommendations definition for the proper decision-making process performance**

The obtained results from Level 4 are the basis for defining the main recommendations for a decision-maker. What is also essential is how strongly the results of Level 5 and decisions are taken depend on how deep the so-called “risk appetite” of managers will be, which determines risk perceptions. The risk perception of decision-makers determines the value of acceptable risk in machine maintenance processes. In some mines, the acceptance level will be set on a “Significant risk” line, while in others, the acceptable acceptance level will be set only at “Low risk”. Research indicates that risk perception depends on many characteristics of decision-makers, such as age, gender, and education [71]. Therefore, it is recommended that risk analysis teams should be composed of various representatives of the company.

First, based on the obtained risk scores, the decision-maker should also be able to interpret the obtained values correctly. Table 6 describes the possible risk levels in a proposed 4-grade scale.

With this understanding of the risk score, it is possible to identify basic recommendations. The main recommendations based on risk level evaluation can be defined, considering the aspects of maintenance, safety, and resource availability/allocation. This approach will allow a comprehensive view of the mine maintenance problem.

For analyses where the risk ratio assumes values accepted by managers, the most common maintenance recommendations are the following maintenance policies, defined by a producer/implemented in a company. From a safety point of view, it is sufficient to introduce risk-reporting (periodic). Additionally, there is no need to introduce any changes to the current inventory policy; managers should also base their decisions on the available resources.

When the obtained level of risk ratio is higher than the acceptable level, the proposed recommendations should satisfy reliability and safety assumption criteria. For the scenarios where the risk level is at least “Significant,” it is necessary to change the machine maintenance standard. The scope of these changes depends on the value of the estimated risk. In case of a “Significant” threat, the changes usually concern a revision of the inspection interval (e.g., shortening the time between periodic inspections) or increased attention to certain elements during the current inspection performance (daily machine maintenance). However, in the case of scenarios with a “High” or “Critical” risk level, the actions taken to reduce this risk should concern not only periodic inspections intervals revision but should also redefine the scope of the condition monitoring and maintenance work carried out. Those recommendations focused on safety and resource allocation/availability should also be carefully developed and implemented. The appropriate recommendations in these two areas will depend on the type of organization (mine type), its physical assets and should be compatible with ISO 5500x standards indications.

**Figure 9:**
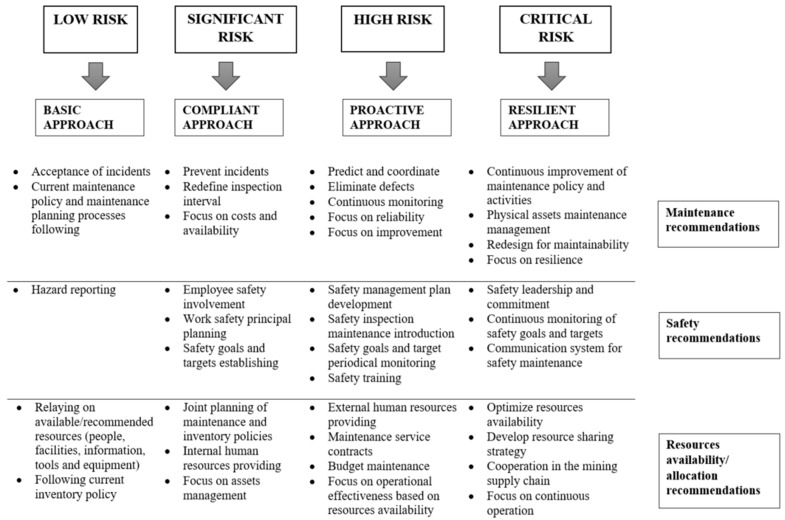
Proposed recommendations, based on obtained risk level—possible directions of company’s related tasks in the three main improvement areas.

Following this, the general areas for recommendations may be compatible with the safety levers presented in, e.g., [6]. The defined safety levers are compared with the assessed risk level (Table 7). Additionally, the general recommendations in relation to the three defined issues—maintenance, safety, and resources availability—are presented in Figure 9. The most crucial improvements are indicated according to the obtained risk level. At the same time, it should be remembered that these recommendations should be considered in terms of three dimensions: persons, system, and machinery.

## 4. Application of the Proposed Approach in a Company in the Mining Industry

To illustrate the implementation possibility of the proposed fuzzy-based decision method, the authors analyzed the process of operation and maintenance of one selected system of mining machinery that has been in operation for three years. The available accident and maintenance data are mainly collected from the maintenance management system’s reports.

To follow the proposed approach, the analyzed adverse event scenarios have been identified and linguistically scored by the experts (underground mining machinery operation and maintenance specialists). The judgment of experienced maintenance experts has been recorded using a survey questionnaire. Maintenance experts have rated each scenario for its probability (according to the decision algorithm presented in Figure 4) and consequences (according to a defined class of attention). These ratings have been based on the linguistic scales presented in Table 2 and Table 3. The identified adverse event scenarios with linguistic scores are shown in Table 8.

**Figure 10:**
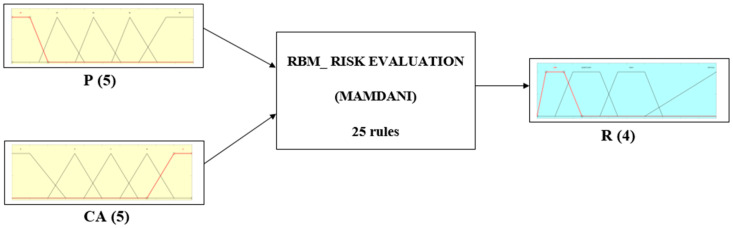
Structure of the proposed fuzzy model.

**Figure 11:**
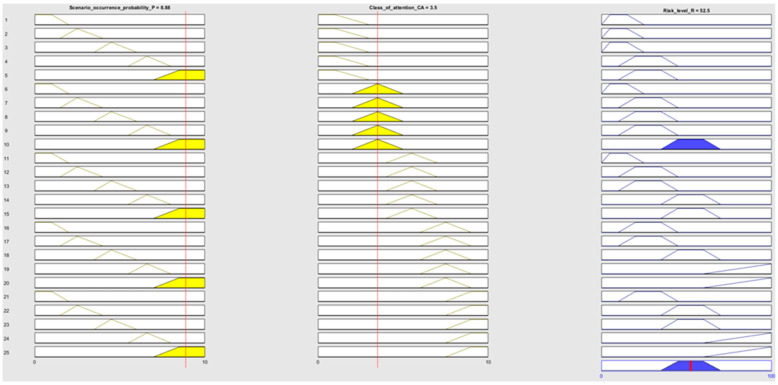
Sample rule base for the proposed fuzzy decision-making approach.

In the next step, the linguistic scores given by the experts have been converted to corresponding fuzzy set numbers (based on Table 4) to be used as an input for the fuzzy inference system in the next step. Using the developed Mamdani-type fuzzy model (Figure 10), it has been possible to obtain the risk score (crisp output value) for all the identified adverse event scenarios, as shown in Table 9. The results in Table 9 are obtained, considering the assumptions that all decision rules have the same weight (the same importance). A sample rule base is presented in Figure 11, and the discussion of obtained results is shown in the next section.

## 5. Results and Discussion

The proposed case study is given to present the implementation possibilities of the developed fuzzy-based approach in the maintenance decision-making process. Moreover, it provides a simple method for risk level assessment based on the use of fuzzy sets. Following the obtained results and implementation procedure, our analysis indicates that it is easier to express the risk as a linguistic term than to present it as a numerical value. Applying the fuzzy logic theory provides a broader picture of the behavior or tendency of failure.

**Figure 12:**
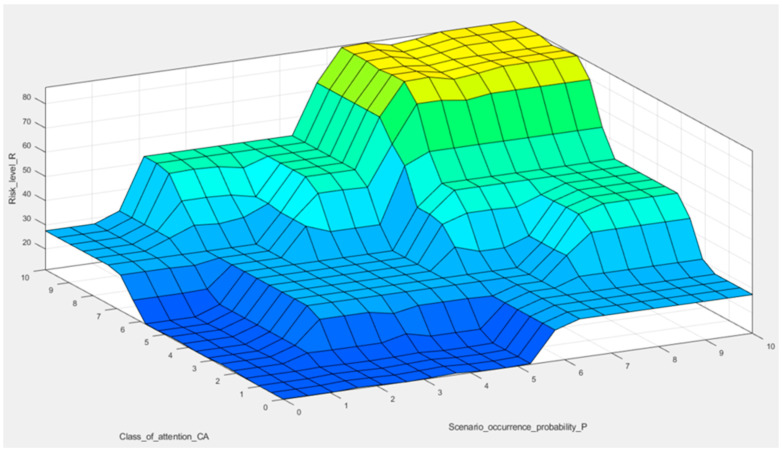
Surface view of the proposed fuzzy inference system for mining machinery (rules with weights equal to 1).

The complete results of the proposed FIS for risk ratio assessment are presented in Figure 12. The 3D plot helps to examine the consistency of the rules framed in the suggested FIS, by displaying the dependency of the output as a function of the inputs.

Following the results of the 3D plot (Figure 12), the constructed FIS can make a more realistic evaluation of the risk parameters (P and CA) by supplying gradual transitions in risk scores and consistent risk categories. The lowest, dark blue part of the plot represents the resultant low level of risk R, resulting from low scenario occurrence probability and the class of attention levels or lack of regularity of process performance, allowing for disruptive event identification, processes monitoring, or forecasting. This reflects the most favorable situation, in which the risk is low and does not require significant changes to mining companies’ operational and management processes.

It should be mentioned that the uppermost corner, the yellow field, represents theoretically the highest score—the critical level of risk in an organization, which would require immediate decisions to minimize risk in relation to maintenance planning processes, safety aspects, and the availability of resources in the organization.

Therefore, the presented flowchart can be used in the process of managerial decision-making regarding active methods aimed at reducing the level of risk, or assessing whether the decisions taken will have the expected effect. This will facilitate the development of an effective risk management plan focused on taking preventive actions for the most risky or disruptive events. Thereby, the organization’s safety can be improved.

According to the obtained results for the investigated mining machinery, 3 adverse-event scenarios have been assessed critically. As a result, it is clear that a maintenance manager should first identify actions that will reduce the level of risk in terms of available technical, human, infrastructure, and organizational resources. For instance, the recommendation can be proposed so that the maintenance resource can be reasonably allocated. The maintenance budget can be planned according to the maintenance cost factors of asset failures. The spare assets can be used according to operational flexibility requirements in relation to the accepted risk level. The safety and environmental impacts of the risks in the process operations of a machine can be reduced by considering the management planning prioritization according to the levels of the safety requirements and environmental impact of the investigated machinery. The general recommendation may include:

- Re-planning maintenance processes (maintenance strategies) to help achieve the required availability, reliability, and safety levels dictated by the business;
- Developing guidelines for the forecasting process, preventive procedures, and maintenance management scenarios for identified adverse events scenarios;
- Assessing strategy elements aimed at achieving the required availability, reliability, and safety levels dictated by the business (e.g., critical spare management, operational controls, and failure response measures);
- Being compliant with statutory and regulatory imperatives (e.g., in safety aspects).

One important aspect of preventive actions supporting the risk-based maintenance concept is equipping mining machines with appropriate sensors, monitoring the current operation of the machine, and reporting permissible values when they are exceeded. The data coming from sensory measurement systems should feed the knowledge base used in the conducted risk analysis, especially in the second step of the proposed analytical procedure. When a machine is monitored primarily by its operator, some signals of abnormal operation can be ignored or disregarded if they do not significantly interfere with operational activities. Therefore, the person performing the risk analysis may not have complete operational data. The constant sensory monitoring of machine operation will make it possible to register any deviations that occur. This provides the analyst with a true overview of the machine’s operation, which increases the accuracy of predicting undesirable events. Examples of sensors that can be used in the operation of the analyzed machine are vibration measurement devices used to detect changes in, e.g., motors and gearboxes. Additionally, pressure sensors can be used with a temperature-compensated output or onboard toxic gas detection modules.

## 6. Conclusions

Maintaining a machine’s efficiency and quickly recovering function in the event of failure plays a key role in managing technical systems. Every day or even hour that a machine is unavailable can translate into significant financial losses for a company. However, this current time pressure to complete tasks cannot be the main guideline when defining maintenance procedures for this machine. It is also not possible to base maintenance decisions for all machines solely on existing standards. Each machine is operated in a different working environment that may significantly impact its technical condition. Both of these factors may lead to machinery that is not fully operational, which, in the process of intensive work, may become even more damaged or even endanger the lives or health of the operators or other crew members.

Therefore, the authors herein proposed an approach in which maintenance recommendations for a given machine are based on the estimated risk of certain adverse events. By using the risk analysis results, decision-makers can make faster and more effective decisions about the maintenance process, considering maintenance standards and machine operating conditions and data from measurement systems. The proposed approach also avoids the occurrence of adverse events with serious consequences, such as the loss of health or the life of crew members. By implementing preventive procedures for high-risk events, managers can secure the system in advance against the occurrence of these events. This plays a particular role in human–technical systems such as underground mines, where the number of factors that threaten the health and lives of miners is much higher than in traditional systems.

Additionally, the proposed approach also takes into account safety aspects in the decision-making processes. The authors introduce possible safety recommendations to ensure the efficient and safe operation of mines. The aspect of resources availability also supplemented the establishment of recommendations. Considering these three aspects—maintenance, safety, and resources—will enable a company’s effective and deliberate management in the mining sector.

The fuzzy logic theory implementation provides a clearer picture of risk-level perception in the domain of expert assessment. This approach is easy to understand and use for non-specialists. Additionally, the proposed methodology tries to include the complete factors that influence process operations and provides complete feedback for a decision-maker in the form of the most important recommendations in the fields of maintenance, safety, and resource management. However, one of the limitations of the proposed approach is connected with fuzzy membership function development. The proposed combination of FNs is not universal, and some other forms of membership functions may be required in other applications. The definition of which membership functions are necessary for other implementation cases will depend on the experts’ knowledge of fuzzy logic theory. It is also essential that the successful and accurate application of the proposed methodology depend on membership function and IF-THEN rules. Therefore, the authors recommend performing several attempts and modifications to ensure a proper risk analysis for any other practical situation. Fortunately, the proposed solutions of fuzzification and defuzzification processes can be employed. Even if other fuzzy membership functions are incorporated, the model can also be modified to accommodate this possible issue.

Another interesting option is to investigate the problem of feedback loops in the developed five-level decision-making method. On the one hand, such feedback analysis is valuable for fault diagnosis and reliability analyses when performed in dynamic systems. On the other hand, this is a part of continuous learning approach implementation. The classical approach, based on fuzzy set theory, does not address this problem. Therefore, the attempt to develop the developed approach with so-called feedback analysis may be an interesting direction for future research work in this area. One of the options could be to evaluate the possibility of using the fuzzy cognitive map (FCM)-based approach.

Moreover, the introduced method enables the more effective incorporation of expert knowledge during risk ratio estimation. Based on the professional opinions of personnel, who have relevant awareness about the different possible failure and hazard scenarios, it is possible to incorporate real circumstances during the maintenance decision-making process. As a result, one application possibility is to link the suggested approach to an existing maintenance management system available in the mining company.

The proposed concept for the prepared recommendations was developed within the framework of the SafeMe4Mine project. The project is carried out with the help of an international team comprising representatives of research centers, machinery manufacturers, and mines. As a result, the methodology we developed takes into account current scientific research results in this area and the experience and opinions of practitioners involved in operating these machines.

Additionally, the proposed approach was verified based on the experience of the companies cooperating in the project consortium. These companies specialize in the manufacturing and operation of drilling machines. Therefore, the proposed approach in the presented scope can be applied in underground mines operating this type of equipment. However, the approach itself is universal and, after adaptation (e.g., in terms of experts’ assessment, description of recommendations for risk levels), it can be applied in any mine for the assessment of any type of machine. However, one must be aware that this requires careful revision of the assumptions made.

In subsequent research work, the proposed methodology will be further developed to improve the results obtained. One of the planned directions of development work is to apply multi-criteria decision-making methods to select the most important criteria from the investigated company safety aspects. The proposed approach is based on the use of the AHP method and provides a methodology better adapted to the needs of the mine considered. Another possible direction of future work is the development of resource allocation strategies that focus on maintenance problems and that are compatible with the requirements of ISO 5500x standards. It is also essential that further research should be carried out to establish an efficient and effective approach to developing membership functions with the help of data-driven models, such as artificial neural networks.

## Figures and Tables

**Table 2:** Assigning probability levels to linguistic notations.

Probability Level	Linguistic Notation	Description
P1	RARE	Could happen but practically impossible that it occurs in the near future
P2	UNLIKELY	Not likely to occur in normal circumstances; conceivable but possible
P3	POSSIBLE	May occur in normal circumstances; unusual but possible
P4	LIKELY	Expected to occur at some time; quite possible
P5	ALMOST CERTAIN	Expected to occur regularly under normal circumstances; may be expected

**Table 3:** Class of attention with consequences description.

Class	Human	Machinery	Costs
A	Injuries and fatalities; permanent total disability	Long-term machine shutdown (more than one month) or permanent withdrawal from an operation	Catastrophic financial losses; very high costs connected with machine shutdown influencing production disruptions, lack of resources, the need to buy a new machine, damage to property, and a significant loss of reputation for the company
B	Major injury, requiring long-term treatment and therapy	Long-term machine shutdown (exceeding three working days)	High costs associated with the shutdown and restoration of a system; high costs of production delays, asset allocation, expenses related to damage to the company’s property and its reputation
C	Minor injury; requiring inpatient treatment	A short-term shutdown lasting not longer than three days	Significant costs associated with shutdown; noticeable costs of production delays, consumption of assets, and damage to the company’s reputation
D	No casualties; requiring outpatient treatment without a lasting impact and requiring first aid	Minor shutdown; no longer than 24 working hours loss	Low costs, mostly connected with the production delay
E	No casualties; no requirement for first aid	Insignificant shutdown; no longer than the eighth loss of working hours	No or insignificant costs

**Table 4:** The numerical value of the linguistic P, CA, and R variables.

Linguistic Variable: Probability P		
**Linguistic Notation**	**Linguistic Value**	**Numerical Range**
P1	RARE	[0, 1, 2]
P2	UNLIKELY	[1.5, 2.5, 4]
P3	POSSIBLE	[3.5, 4.5, 6]
P4	LIKELY	[5.5, 6.5, 8]
P5	ALMOST CERTAIN	[7, 8.5, 10, 10]
Linguistic variable: class of attention CA		
**Linguistic notation**	**Linguistic value**	**Numerical range**
A	VERY HIGH	[7.5, 9, 10, 10]
B	HIGH	[6, 7.5, 9]
C	MEDIUM	[4, 5.5, 7]
D	LOW	[2, 3.5, 5]
E	VERY LOW	[0, 0, 1, 3]
Linguistic variable: risk R		
**Linguistic notation**	**Linguistic value**	**Numerical range**
L	LOW	[0, 5, 15, 25]
S	SIGNIFICANT	[10, 20, 35, 45]
H	HIGH	[35, 45, 60, 70]
CR	CRITICAL	[60, 100, 100]

**Table 6:** Risk levels and scores.

Ranking Category	Description	Risk Sacore
CRITICAL	The probability of an adverse event occurring is almost certain, with severe consequences in terms of long-term machine downtime and possible personnel casualties, such as injuries or fatalities.	81–100
HIGH	The adverse event has a high probability of occurrence, but its effects affect the operation of the machine in the medium term; the possible shutdown does not exceed three days. Alternatively, the event generates high losses in the form of long-term machine shutdown and even injuries, but the probability of its occurrence is low.	46–80
SIGNIFICANT	Adverse events are characterized by an average probability of occurrence and a potential consequence. If an extreme value of one of the evaluated parameters occurs, the value of the other parameter takes the opposite extreme value (e.g., very high probability—very low effect).	26–45
LOW	The adverse event risk is very low and, at the same time, the consequences of its occurrence do not significantly affect current mining processes.	0–25

**Table 7:** Main areas for recommendations based on the obtained risk level.

Main Area for Recommendations	Risk Level			
	LOW	SIGNIFICANT	HIGH	CRITICAL
Regulatory	x	x	x	x
Economic		x	x	x
Organizational/managerial		x	x	x
Operational/maintenance		x	x	x
Technical/design				x
Research/education	x	x	x	x

x—should be applied.

**Table 8:** Parameters linguistic scores for all identified adverse event scenarios, based on experts’ opinions.

No.	Adverse Event Scenario	Scenario Occurrence Probability P	Class of Attention CA
1	Faulty fire suppression system	P3	B
2	Leakage in the hydraulic piping system	P5	B
3	Faulty power train	P4	B
4	Faulty combustion engine in the drive train	P3	B
5	Faulty break brake system	P4	A
6	Faulty electric drive of the working hydraulics	P3	B
7	Hydraulic pump failure in the hydraulic system	P3	C
8	Failure of the air intake system in the drive train	P3	C
9	Failure of the boom in the working system	P4	D
10	Failure of the air conditioning system	P3	D
11	Electrical failure in the electrical system DC 24 V	P3	C
12	Electrical failure in the electrical system AC 500/1000 V	P3	A
13	Failure of the machine frame, which is a structural element	P2	B
14	Failure of the drilling machine, which is part of the working system	P5	D

**Table 9:** The risk score and risk level of identified adverse event scenarios.

No.	Adverse Event Scenario	Risk Score	Risk Level
1	Faulty fire suppression system	52.5	HIGH
2	Leakage in the hydraulic piping system	87	CRITICAL
3	Faulty power train	87	CRITICAL
4	Faulty combustion engine in the drive train	52.5	HIGH
5	Faulty break brake system	87	CRITICAL
6	Faulty electric drive of the working hydraulics	52.5	HIGH
7	Hydraulic pump failure in the hydraulic system	27.5	SIGNIFICANT
8	Failure of the air intake system in the drive train	27.5	SIGNIFICANT
9	Failure of the boom in the working system	27.5	SIGNIFICANT
10	Failure of the air conditioning system	27.5	SIGNIFICANT
11	Electrical failure in the electrical system DC 24 V	27.5	SIGNIFICANT
12	Electrical failure in the electrical system AC 500/1000 V	52.5	HIGH
13	Failure of the machine frame, which is a structural element	27.5	SIGNIFICANT
14	Failure of the drilling machine, which is part of the working system	52.5	HIGH

## Data Availability

Not applicable.

## Abbreviations

AHP	Analytic Hierarchy Process
DEMATEL	Decision Making Trial and Evaluation Laboratory
DMRA	Decision Matrix Risk-Assessment
ETA	Event Tree Analysis
FIS	Fuzzy Inference System
FLS	Fuzzy Logic System
FMECA	Failure Mode, Effects and Criticality Analysis
FN	Fuzzy Number
FRA	Fuzzy Reasoning Approach
FTA	Fault-Tree Analysis
HAZOP	Hazard Operability Study
MCDM	Multiple-Criteria Decision-Making
PRAT	Proportional Risk Assessment
QRA	Quantitative Risk Assessment
RBIM	Risk-based Inspection and Maintenance
RBM	Risk-based Maintenance
RPN	Risk Priority Number
SWIFT	Structured “What If” Technique
TOPSIS	Technique for Order of Preference by Similarity to Ideal Solution
WRA	Weighted Risk Analysis
